# Reply to: “The diamine cation is not a chemical example where density functional theory fails”

**DOI:** 10.1038/s41467-018-07683-z

**Published:** 2018-12-17

**Authors:** Xinxin Cheng, Elvar Jónsson, Hannes Jónsson, Peter M. Weber

**Affiliations:** 1 Department of Chemistry, Brown University, Providence, RI 02912 United States; 2 Faculty of Physical Sciences, VR-III, University of Iceland, 107 Reykjavík, Iceland; 3 The Hamburg Centre for Ultrafast Imaging (CUI), Universität Hamburg, Luruper Chaussee 149, 22761 Hamburg, Germany; 4 Max Planck Institute for the Structure and Dynamics of Matter (MPSD), Luruper Chaussee 149, 22761 Hamburg, Germany

**Replying to** Z. A. Ali et al. *Nature Communications* 10.1038/s41467-018-07266-y (2018)

In their correspondence related to our article on the localized and delocalized electronic states of N,N’-dimethylpiperazine (DMP)^1^, Ali et al.^2^ present interesting new calculations that complement the results we had presented. None of the calculations they present contradict our results, but provide additional information about the coupled cluster singles-doubles (CCSD) energy surface, including the first order saddle point that gives an estimate of the activation energy barrier for the transition between the two states, and some information about the CCSD(T) (coupled cluster with single and double excitations and perturbative triples) energy surface where the effect of connected triple excitations has been added in a perturbative way. Configurations are given for the two states, as well as the first orders saddle point configuration at the Møller-Plesset second order (MP2) perturbation theory level, but the calculated activation energy is not reported. While the data presented in the correspondence of Ali et al. is valuable, we do not agree with all their interpretations, as we discuss below. The key issue is the energy barrier in the transition from the localized to the delocalized state of the DMP cation.

Our experimental measurements clearly show that an energy barrier exists between the localized and delocalized DMP Rydberg states. Without it we would not be able to identify the localized state. While the possibility of coherent dynamics prevailing over kinetics is real and has been observed in other systems^3,4^, the time resolved spectra displayed in Fig. 1 of ref. ^1^ are consistent only with a kinetic transition. A dynamic transition would lead to a continuous evolution between the early-time and the late-time peaks, which is not observed. Moreover, the time scales involved in the transition from the localized state to the delocalized state is well into the picosecond range^5^ and can only be consistent with a kinetic transition. Assuming a standard pre-exponential factor, the observed time scale of picoseconds indicates there is an energy barrier on the order of 0.1 eV. While it is true that the time dependence of the kinetics was measured in the Rydberg state rather than the ion state, we do not think this will make an essential difference because the potential energy surfaces of Rydberg states are typically very similar to those of the corresponding ion states. In the present system we observe no vibrational progression, showing that the surfaces are indeed very similar.

Consistent with our presentation of a metastable localized state at the CCSD level of theory^1^, Ali et al. indeed find an energy barrier for the transition to the delocalized state. The fact that the energy barrier then disappears when the effect of connected triples excitations is included in a perturbative manner in the CCSD(T) method is interesting. One issue is the need for a multireference description of this system. Ali et al. carried out the T1 test of multireference character and obtained values as large as 0.031^2^. Experts in the field have specified that a multireference description is indeed needed when this value exceeds 0.02^6^. A better test of the accuracy of the CCSD(T) approximation is to carry out calculations at a higher, multireference level of theory. This has recently been done (H.J., manuscript in preparation) and we quote the key results here. The calculations are carried out at the complete active space self-consistent field (CASSCF) level of theory formulated within the density matrix renormalization group (DMRG) method^7,8^. The active space includes 19 electrons and 20 orbitals represented by the aug-cc-pVTZ (augmented correlation-consistent polarized valence triple-zeta) basis set^9^ (chosen to give convergence for both the localized state and the saddle point). The ORCA software was used for the calculations^10^. An energy barrier of 0.072 eV was obtained which is in agreement with the rough experimental estimate described above. The calculated CCSD(T) results presented by Ali et al. suggesting no energy barrier therefore indicate a failure of the CCSD(T) approximation in describing the transition state in this case. It is in fact well known that CCSD(T) often fails for transition states^11^.

We emphasize that some density functional theory (DFT) calculations do predict an energy barrier for the transition. As pointed out in ref. ^1^, the half-and-half hybrid functional of Becke, referred to as BHandHLYP, supports a metastable localized state. The functional contains 50% exact exchange as deduced from arguments based on the adiabatic connection formula^12^. This is, however, not a commonly used functional in comparison with, for example, B3LYP where the fraction of exact exchange is reduced to 20% in order to fit more closely various thermochemical data on molecules. While the reduction of the weight of exact exchange can provide a better fit to some properties, other properties can be less well reproduced, for example transition states for electron transfer reactions^13^. The BHandHLYP energy barrier has recently been calculated and was found to be 0.033 eV (H.J., manuscript in preparation). If the weight of exact exchange is increased, the energy barrier becomes larger. With 70% exact exchange the barrier is 0.19 eV (H.J., manuscript in preparation). The lack of a metastable localized state for the DMP cation with commonly used DFT functionals is, therefore, not a limitation of the DFT approach, but rather the functionals that have been fitted, and possibly overfitted, to thermochemical data sets. Functionals with lower weight of exact exchange include larger self-interaction error than functionals with higher weight. Explicit Perdew–Zunger self-interaction correction (PZ-SIC) is known to increase energy barriers as compared with commonly used DFT functionals^14^.

We note also that the MP2 level of theory does predict a significant energy barrier for the transition, 0.13 eV (H.J., manuscript in preparation), even though the product state, the delocalized state, is much too low in energy compared with the localized state, as we presented in ref. ^1^.

Ali et al. point out that the saddle point configuration reported in our article for the PZ-SIC calculation lacks symmetry in the orientation of a CH_3_- group. The reason for this is a loose convergence criterion in the saddle point calculation presented in ref. ^1^. The tolerance on the atomic forces was in that case set to 0.05 eV/Å, a value that is typically used in condensed matter calculations. Calculations with, for example, the BHandHLYP functional also show analogous lack of symmetry with this tolerance, but with a tighter tolerance of 0.005 eV/Å the symmetry is restored (H.J., manuscript in preparation). This, however, does not have a significant effect on the energetics. Such deviation in the orientation of the CH_3_- group involves only a tiny change in the energy. The Perdew–Zunger self-interaction calculations are being carried out with a tighter tolerance and improved optimization algorithm (E.J., H.J., manuscript in preparation). The calculations are more challenging than regular DFT calculations because of the explicit orbital density dependence^15,16^. In more important respects, the geometries reported in ref. ^1^ from the PZ-SIC calculations are in remarkably close agreement with the CCSD and CASSCF/DMRG calculations, closer than the BHandHLYP configurations. For example, one of the N-C…C-C dihedral angles is 134° in BHandHLYP, but 102° in PZ-SIC^1^, 103° in CCSD^2^, and 105° in CAS-SCF/DMRG (H.J., manuscript in preparation) for the localized state.

In conclusion, the calculations presented in the correspondence by Ali et al. on the DMP cation are interesting and make a valuable addition to the calculations we presented in ref. ^1^. The DMP cation remains an interesting system for testing theoretical methodologies, such as different energy functionals and quantum chemistry approximations. The interpretation of the calculations presented in the correspondence by Ali et al. is, however, neither consistent with results of the experiments nor higher level, multireference calculations (H.J., manuscript in preparation).

## Code availability

Custom computational codes of this study are available from the corresponding author upon reasonable request.

## Data Availability

All relevant data of this study are available from the corresponding author upon reasonable request.
